# Vision-Based Tunnel Lining Health Monitoring via Bi-Temporal Image Comparison and Decision-Level Fusion of Change Maps

**DOI:** 10.3390/s21124040

**Published:** 2021-06-11

**Authors:** Leanne Attard, Carl James Debono, Gianluca Valentino, Mario Di Castro

**Affiliations:** 1 Department of Communications and Computer Engineering, Faculty of ICT, University of Malta, MSD 2080 Msida, Malta; leanne.attard@um.edu.mt (L.A.); gianluca.valentino@um.edu.mt (G.V.); 2 Engineering Department, SMM Group, CERN, CH-1211 Geneva, Switzerland; mario.di.castro@cern.ch

**Keywords:** computer vision, data fusion, tunnel lining inspections

## Abstract

Tunnel structural health inspections are predominantly done through periodic visual observations, requiring humans to be physically present on-site, possibly exposing them to hazardous environments. These surveys are subjective (relying on the surveyor experience), time-consuming, and may demand operation shutdown. These issues can be mitigated through accurate automatic monitoring and inspection systems. In this work, we propose a remotely operated machine vision change detection application to improve the structural health monitoring of tunnels. The vision-based sensing system acquires the data from a rig of cameras hosted on a robotic platform that is driven parallel to the tunnel walls. These data are then pre-processed using image processing and deep learning techniques to reduce nuisance changes caused by light variations. Image fusion techniques are then applied to identify the changes occurring in the tunnel structure. Different pixel-based change detection approaches are used to generate temporal change maps. Decision-level fusion methods are then used to combine these change maps to obtain a more reliable detection of the changes that occur between surveys. A quantitative analysis of the results achieved shows that the proposed change detection system achieved a recall value of 81%, a precision value of 93% and an F1-score of 86.7%.

## 1. Introduction

Tunnel infrastructure shows signs of deterioration over time due to construction defects, ageing, unexpected overloading and natural phenomena, possibly leading to problems in structural integrity. Consequently, periodic inspections of concrete tunnels should be conducted to ensure that they are still healthy and safe. Today, these are predominantly performed through periodic visual observations, looking for structural defects such as cracking, spalling and water leakage to identify possible changes in the infrastructure with respect to a previous survey. Strain gauges, displacement meters and other contact measurement methods can be employed to monitor problematic areas more closely within these structures. To conduct such observations, personnel are required to be physically present in the tunnel. Associated with this, there are several drawbacks, including the human presence in hazardous environments and the financial cost involved to train and hire people to do the inspections. In addition, these inspections require a considerable amount of time to perform, leading to longer operation down-times and thus higher monetary losses. Furthermore, the outcome from these inspections is highly dependent on human subjectivity, leading to possible inaccuracies that result in false and missing change detections.

All this has led to an increase in the demand for robotic systems and remote operations to reduce direct human intervention. One possible solution is to use vision-based sensing monitoring systems that can provide reliable objective results. Hence, a substantial effort can be found in the literature on automating inspections using image processing and machine learning methods to detect and classify cracks, structural deformities, and the presence of water along the tunnel linings. Moreover, the imagery can also be used to provide multiview point clouds to visualise the location of these defects and identify issues with surface deformation [1,2]. This task is not trivial as tunnel environments are characterised by non-uniform illumination and shadows, deformations, lack of features, dirt, stains, and possible occlusion of parts of the walls due to cables, pipes and other servicing equipment.

Whilst defect identification is essential to automate inspection, regular monitoring of tunnel linings can provide a more informative survey to further automate inspection and analysis. In this paper, we present a tunnel inspection application that uses robotics, computer vision and data fusion to monitor for changes on tunnel linings. The main contributions of this work are:

- Integration of a commercial inspection camera system on a robotic platform;
- Development of a specular highlight localisation algorithm based on uneven illumination correction and deep learning to remove these artefacts from the images captured by the system;
- Implementation and analysis of bi-temporal image fusion techniques for image comparison and change-map generation;
- Implementation and evaluation of two decision-level fusion techniques for robust change detection.

The tunnel used to develop this system is within CERN, the European Organisation for Nuclear Research. The considered tunnel is a 27 km long tunnel lying at around 100 m below the ground, hosting the world’s largest particle accelerator, the Large Hadron Collider (LHC).

The remainder of this article is structured as follows. Section 2 reviews the state-of-the-art with respect to automated tunnel inspection and the techniques used here. The proposed solution is presented in Section 3. Section 4 explains the image acquisition part. In Section 5, pre-processing tasks are described. Bi-temporal image fusion is described in Section 6. Section 7 discusses decision-level fusion in the context of change detection, followed by the change map (CM) analysis process presented in Section 8. A performance evaluation is made in Section 9. A summary and suggestions for future work conclude this article.

## 2. Background Information

### 2.1. General Tunnel Inspection

Research on automated health monitoring of tunnel structures has received significant attention in recent years, as recorded in [3,4]. Various solutions that deal with different aspects of automated tunnel inspection were proposed through the use of cost-effective photographic equipment and computer vision. In [5], an extensive survey of works within the whole image-based tunnel inspection spectrum is presented. This includes tunnel profile monitoring, crack and leakage detection, as well as tunnel surface documentation and visualisation.

### 2.2. Change Detection

Change detection is a well-researched problem in the fields of video surveillance, remote sensing ([6,7]) and medical imaging, amongst others. Reviews of change identification methods are found in [8,9]. However, literature on the detection of changes on tunnel linings is still lacking, possibly due to the challenges encountered in this area. Some of these can be referred to in [10,11,12,13,14]. A system aimed at supporting structural inspectors to monitor the condition of railway tunnels is presented in [10]. An array of cameras with uniform lighting is used to capture the image data that are registered and stitched to allow tunnel inspectors to examine for defects with reference to their location. The solution presented also implements a change detection algorithm to attract attention to areas that are considered important, and the inspectors can refer to previous imagery from previous inspections to assess the evolution of the defect. A three-dimensional model of the structure is also created to better contextualise these defects. This paper does not report on the accuracy of the techniques used. A change detection system that relies on computer vision techniques was presented in [11]. A camera was implemented on a monorail, and the captured images were first processed by a shading correction algorithm before using image mosaicing to correct for offsets between successive inspections. A change detection algorithm was then applied on a survey image and the reference image using image differencing, binary image comparison and optical flow analysis. The solution in [12] registers new images on a three-dimensional surface model to detect and assist visual inspections. The changes are detected using a probabilistic model that considers different feature maps and a geometric prior to reducing the impact of noise and nuisance sources. The system presented in [13] builds panoramas of the tunnel surface and registers images from different inspections. A two-channel convolutional neural network is used to determine anomalous changes between the current survey image patch and the corresponding reference. The work in [14] discusses the application of Procrustes methods in photogrammetry. A method that uses freeform surface modelling and a mask region-based convolutional neural network is presented in [15] to generate 3D tunnel structures and superimpose the position of cracks on the model. Change detection techniques can be applied to measurements completed at different times.

The goal of any change detection algorithm is to detect significant changes between the new measurement and a reference, where the reference is the previous measurement at the exact same location. However, this is not a trivial task as the accuracy of such algorithms can be hindered by apparent intensity changes that result from camera motion and different lighting conditions. Hence, pre-processing steps involving geometric, radiometric adjustments and semantic segmentation are generally required as a primary stage to provide more robust change detection solutions.

### 2.3. Data Fusion

Data fusion combines data from different methods for increased reliability, higher redundancy and improved identification. Surveys of different fusion architectures are presented in [16,17]. Image fusion is a specific type of data fusion, classified into pixel, feature and decision levels. Image fusion applications can also be categorised by the time, view or modality at which the images are taken. Multiview applications, such as [18,19], fuse images from the same modality but from different viewpoints. Images taken at different times are combined using multi-temporal fusion to detect changes between them or to synthesise images not photographed at a desired time, as in [20,21]. In multi-modal fusion, images coming from different sensors are combined, such as in [22,23].

## 3. Solution Overview

**Figure 1:**
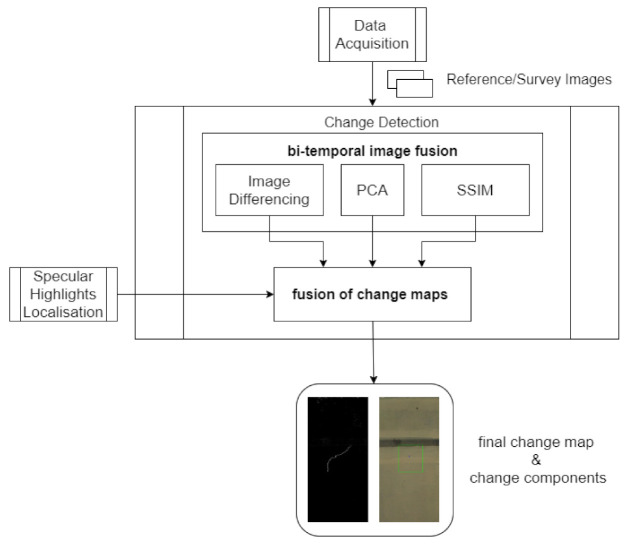
Block diagram of the proposed automatic inspection solution.

The proposed solution is illustrated in Figure 1. The acquisition of images in the tunnel is made by a mobile robotic platform. Pre-processing steps involving radiometric adjustments and specular highlight localisation are applied to minimise false change detections due to camera motion, uneven illumination, and different light reflections. Bi-temporal fusion, involving image differencing, principal component analysis (PCA) and a structural similarity index (SSIM) followed by decision-level image fusion is employed at respective stages to achieve change detection.

## 4. Image Data Acquisition

### 4.1. Acquisition System

**Figure 2:**
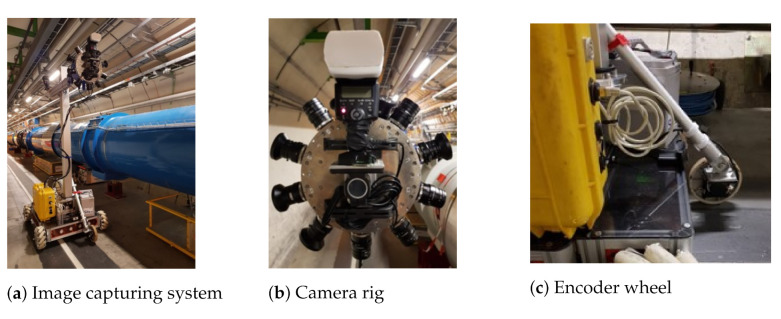
Commercial camera system integrated on the CERNBot. Image capturing system (**a**), camera rig (**b**), encoder wheel (**c**).

A camera system [24] designed to inspect cylindrical environments was identified on the market. The system is composed of a twelve-unit camera rig, as shown in Figure 2b, two flashlights, an encoder wheel, two batteries and a computer unit with software for camera synchronisation. The twelve industrial cameras mounted on the rig had a 5-megapixel resolution with adapted lenses. During a demo test in the LHC tunnel, this system was integrated on CERNBot [25], one of the readily available robotic platforms at CERN, as shown in Figure 2a. The encoder wheel was attached to the CERNBot, as shown in Figure 2c.

### 4.2. Dataset

CERNbot was driven parallel to the tunnel wall at a speed of around 0.2 m/s along a section of the LHC tunnel while capturing synchronised images. This image set is referred to as $DataT_{1}$. Changes to the structure were then simulated by markings on the wall. These markings had different resolutions, with the fine cracks being 0.5 mm wide. The CERNbot was again driven along the same section and at the same speed capturing $DataT_{2}$.

**Figure 3:**
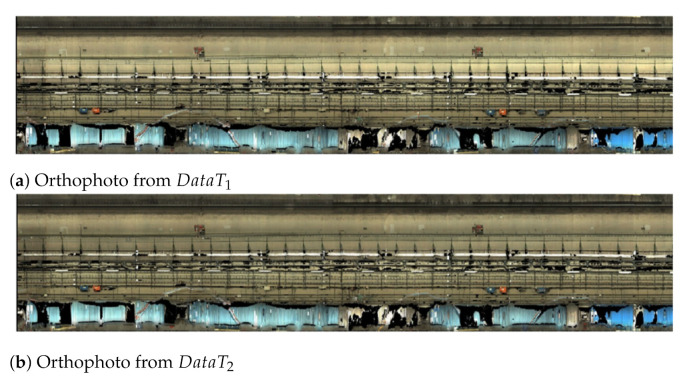
Orthophotos generated from $DataT_{1}$ (**a**) and $DataT_{2}$ (**b**) captured during the demo test.

Using these data, 3D models were generated and unwarped into orthophotos using scripts run by the company supplying the same camera system [24]. Using location information from the encoder wheel, orthophotos could be accurately registered, as seen in Figure 3, such that pixel-based change detection (PBCD) techniques could be applied. Each orthophoto is segmented into ten parts along its height, and each image crop covers 0.5 m of the tunnel length. Such images were used for training and testing of the algorithms used in the proposed tunnel lining change detection solution.

## 5. Image Pre-Processing

The detected changes should be due to new defects or from the evolution of already existing ones. Other changes caused by lighting sources should be identified as “nuisance”, preventing them from being propagated in a change detection pipeline. Hence, pre-processing needs to be completed to cater for this.

### 5.1. Uneven Illumination Correction

**Figure 4:**
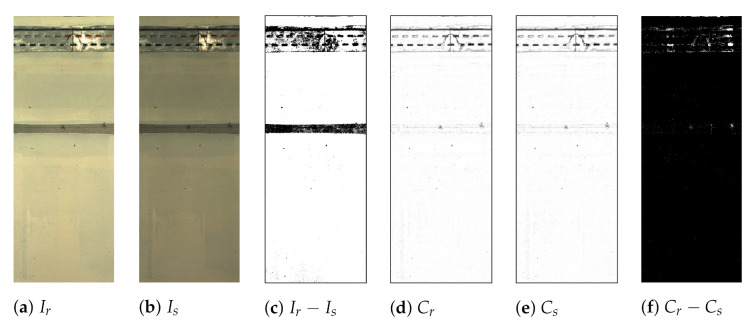
The original reference $I_{r}$ (**a**) and survey $I_{s}$ (**b**) images, the difference image (**c**), the pre-processed reference $C_{r}$ (**d**) and survey $C_{s}$ (**e**) images, and the difference image of the pre-processed images (**f**).

An uneven amount of light falling on different areas causes non-uniformity in images leading to nuisance when comparing images. To adjust the uneven illumination, we use the shading algorithm in [26]. A low-pass filter is applied to the original image using a median filter with a large kernel. The illumination corrected image is obtained through a pixel-wise division of the original image by the low-pass filtered image. As observed in Figure 4c, subtracting the original images generates a difference image full of “white change areas”; however, this is due to uneven illumination. On the other hand, when the images are pre-processed to correct for uneven illumination, their difference image does not have any “white areas” even if there is a change in lighting, as shown in Figure 4f. More details on how this correction mechanism compares to other solutions can be found in [26]. Considering these results, this method proves to be an effective pre-processing method to provide useful images for subsequent processing.

### 5.2. Specular Highlight Localisation

**Figure 5:**
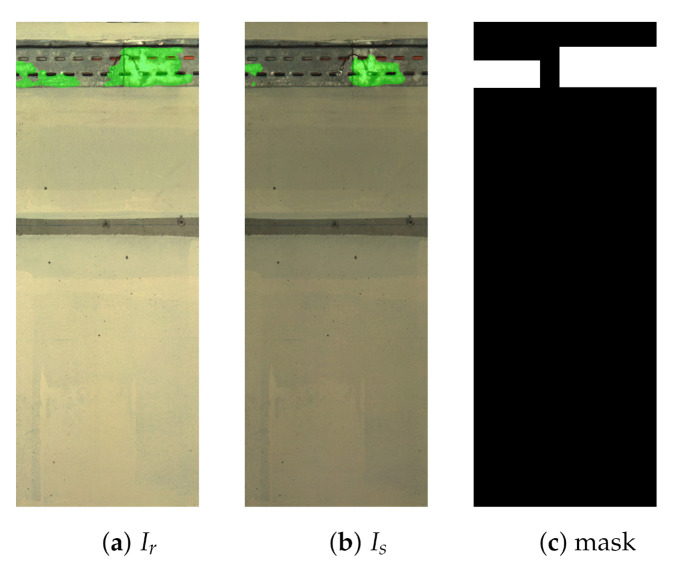
Highlight localisation on the reference image $I_{r}$ (**a**), survey image $I_{s}$ (**b**) and the corresponding highlight mask (**c**).

During image acquisition, flashlights cause reflections on metal racks/pipes present on the wall, resulting in specular highlights in the images. Such highlights are not constant in time or place, leading to false detections when subtracting images to identify changes, as shown in Figure 4. Thus, highlight detection was implemented to localise these regions in the image pair, as displayed in Figure 5. For this, semantic segmentation using a modified U-Net [27] architecture is implemented [28]. Morphological operations and connectivity analysis are then applied to the segmentation images to generate bounding boxes around highlighted areas in the image pair, as illustrated in Figure 5c. Such masks are later fused with the CM to mask out these false change candidates.

## 6. Bi-Temporal Image Fusion

**Figure 6:**
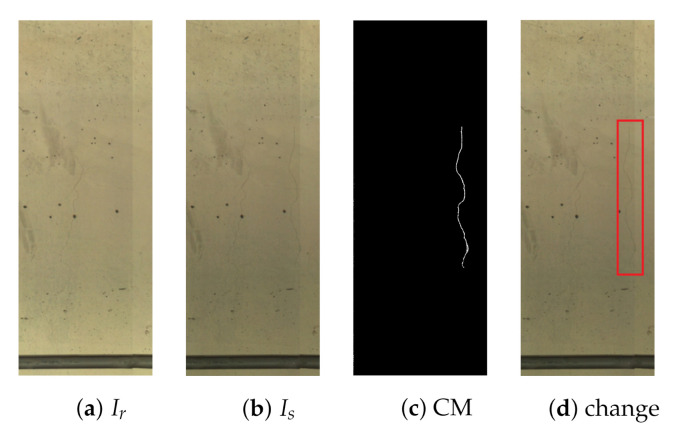
Change detection in an ideal-world scenario. $I_{r}$ (**a**), $I_{s}$ (**b**), CM (**c**) and change (**d**).

Multi-temporal fusion combines data from images of the same scene, acquired at different times. Hence, this approach can be used to identify changes in a scene by comparing images. In this scenario, bi-temporal image fusion is applied between the two temporal images; reference and survey. The reference and survey image pair in Figure 6 is used in the explanation of the subsequent methods.

The image fusion method used in this work involves image differencing, PCA and SSIM. The image differencing method finds the difference between the reference and survey images. In an ideal scenario, the result of this will yield an all-zero image except for where a change occurred. However, errors in registration and in the data acquisition process will depart from this ideal case. The PCA is applied to the stacked reference and survey images. The components of the PCA yield the information from both images, the difference between them and other components representing noise. The component representing the difference is used in this case. The PCA is selected as it removes correlated features focusing on the important features, which are the changes in this case. The final metric chosen is the SSIM, which measures the similarity of the reference and survey images. In this case, we are looking at the parts of the images that are not similar, representing change.

### 6.1. Image Difference

This method has been used extensively for change detection in various applications, including background subtraction for movement detection and remote sensing. As outlined in [29], two images of the same scene taken at separate times $t_{1}$ and $t_{2}$ are subtracted pixel-wise. After the subtraction, the magnitude of the difference value is compared against a threshold. Pixels with a difference magnitude higher than the pre-defined threshold are classified as “change”, otherwise noted as “no change”. The *CM* is generated using:

(1) $$Diff\left(x,y\right)=|I\left(x,y,t_{1}\right)-I\left(x,y,t_{2}\right)|$$

(2) $$CM\left(x,y\right)=\left\{\begin{array}{cc} 1 & \mathrm{if}\;Diff(x,y)\geq T\\ 0 & \mathrm{otherwise} \end{array}\right.$$

where $I(x,y,t_{1})$ is the image at time $t_{1}$, $I(x,y,t_{2})$ is the image at time $t_{2}$ and *T* is the threshold on the difference magnitude. This method is simple and requires low computation; however, its accuracy depends on the threshold set. As *T* is increased, the number of change pixels decreases, implying the elimination of lower difference magnitudes, thus more noise suppression. However, the “valid change” pixels are lost at T≥30 in this particular example.

**Figure 7:**
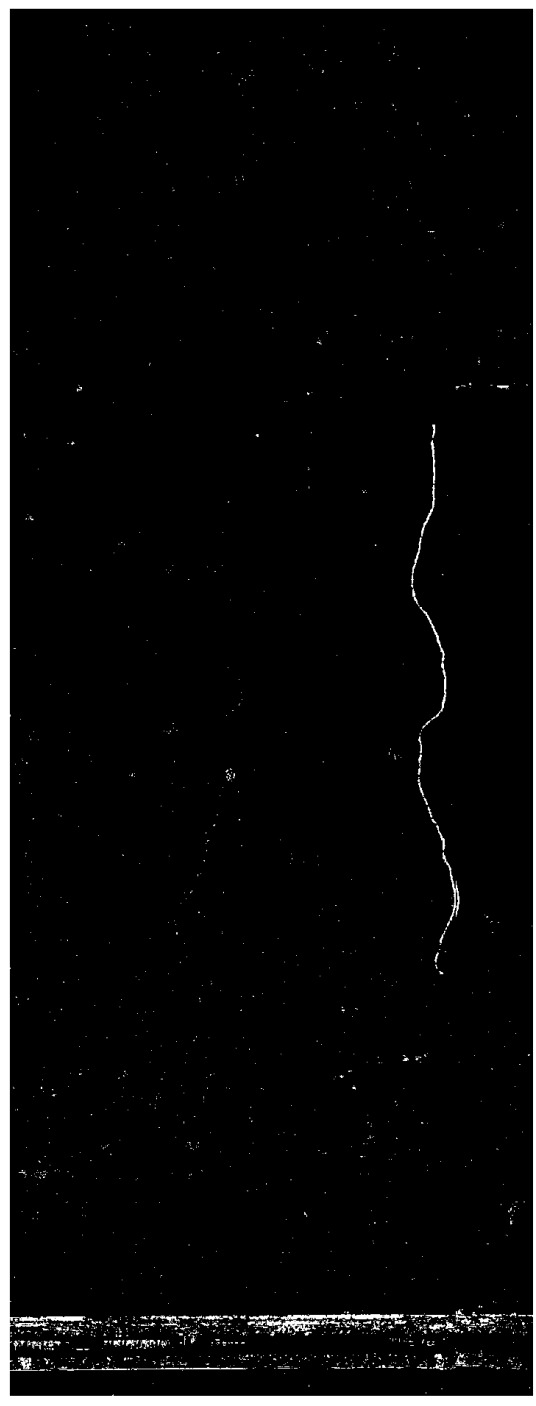
Image differencing using Gaussian valley emphasis for automatic thresholding.

A fixed threshold value cannot satisfy all scenarios; thus, a better approach is to set the threshold automatically depending on the images being compared. The Gaussian valley emphasis (VE) method proposed in [30] is used, generating a CM with only a few “noise changes” while retaining the “crack change”, as observed in Figure 7. The standard deviation of the Gaussian window was empirically set to σ=5.

Approaches such as [31] use multiple frames differencing to mitigate issues of sudden illumination changes and ghosting problems. However, in the tunnel environment, illumination changes at the same locations between surveys are limited and the simple differencing was deemed adequate. Other techniques can be applied to reduce the impact of noise, such as wavelets, where [32] proposed a threshold function that is based in the multi-layer wavelet transform. Moreover, [33] uses wavelets to find a trade-off between the removal of noise and extraction of edges in high-speed moving target imagery.

### 6.2. Principal Component Analysis (PCA)

PCA reduces the dimensionality of a dataset while maintaining the variances. Independent data transformation analysis applies PCA on each of the temporal images separately. The derived principal components are then analysed by applying other change detection techniques, such as image differencing or regression. On the other hand, merged data transformation analysis stacks *N* temporal images of *p* channels each, fuses them into a single N×p-channel image and applies PCA on the latter. In this bi-temporal scenario, the merged data approach is used, and the reference and survey images are stacked on each other. The method was investigated in terms of the original RGB images and the pre-processed images, which is the illumination-corrected images. The processing is done on the RGB data as it provides two more dimensions compared to greyscale images and hence more information that helps in detecting changes.

**Figure 8:**
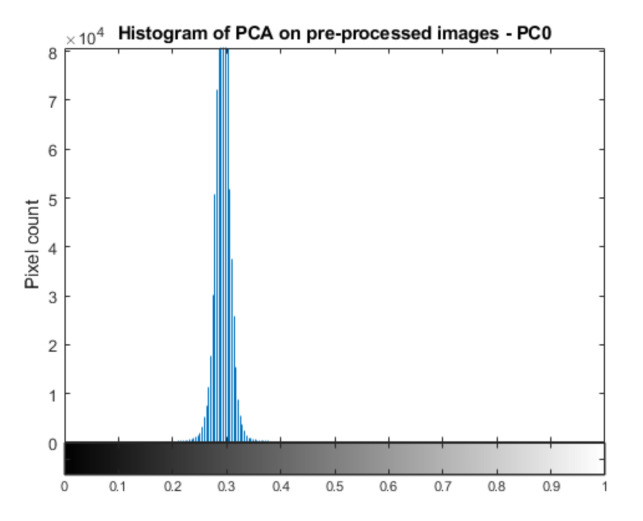
Histogram of normalised $C_{0}$ from PCA on pre-processed images.

When RGB images (p=3,N=2) are used, the stacked images are merged into a 6-channel image. The first component ($C_{0}$), corresponding to the highest eigenvalues, contains most of the information from both images. $C_{1}$ represents the difference between temporal images while later components contain noise information. Experimental results show that PCA is scene-dependent, thus comparison between different data is often difficult to interpret using a fixed condition, implying the need for scenario-dependent thresholds. When considering $C_{1}$, the histogram shape is not clearly defined at its tails, making it difficult to find an adaptive threshold pair. When pre-processed images (p=1,N=2) are used, a stacked 2-channel image is generated. From PCA, the first component $C_{0}$ represents the difference between temporal images while $C_{1}$ contains most of the information from both images. In this case, when considering $C_{0}$, the “crack change” has a high value (white), the “pipe reflections change” has a low value (black) and the rest of the wall has a medium value (grey). This again implies that the histogram contains change elements at both of its tails implying the need for a threshold pair. In this case, however, as observed in Figure 8, the histogram shape follows a Gaussian distribution. To automatically find a threshold pair, the statistical process control (SPC) principle [34] was adopted as it involves binarising an image with a range of pixel values away from the mean pixel where the range is controlled by an input control factor.

The lower and higher thresholds for each image pair under comparison were determined via SPC using:

(3) $$T_{low}=\mu -c\sigma$$

(4) $$T_{high}=\mu +c\sigma$$

where μ, σ are the mean and standard deviation of $C_{i}$, respectively, and *c* is a constant whose value of three was empirically set for the considered dataset.

(5) $$CM\left(x,y\right)=\left\{\begin{array}{cc} 1 & \mathrm{if}\;C_{i}\left(x,y\right)>T_{high}\\ 1 & \mathrm{if}\;C_{i}\left(x,y\right)<T_{low}\\ 0 & \mathrm{otherwise} \end{array}\right.$$

**Figure 9:**
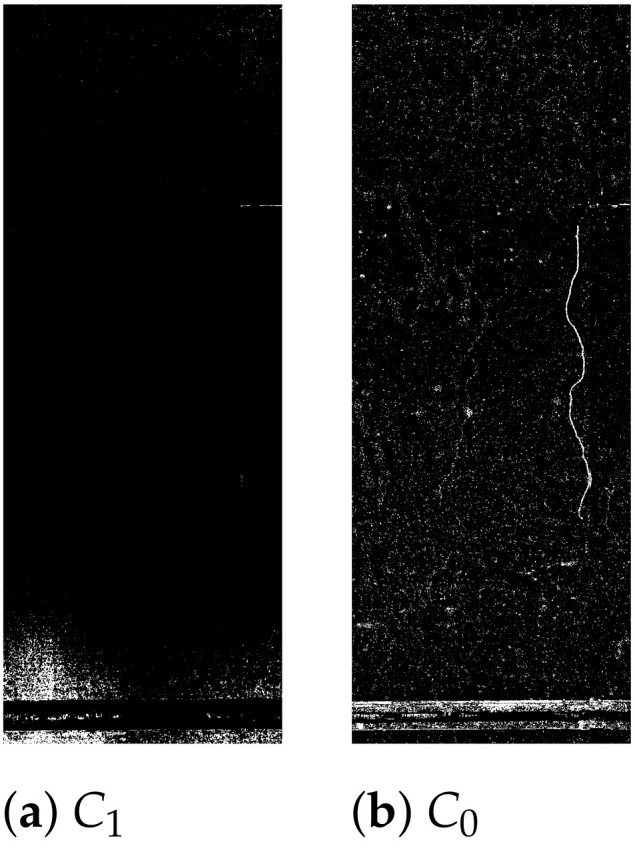
Change maps from PCA applied to different images. $C_{1}$ (**a**), $C_{0}$ (**b**).

Applying Equation (5) on the $C_{1}$ of the original and $C_{0}$ of the pre-processed images generated the CMs in Figure 9.

### 6.3. Structural Similarity (SSIM) Index

**Figure 10:**
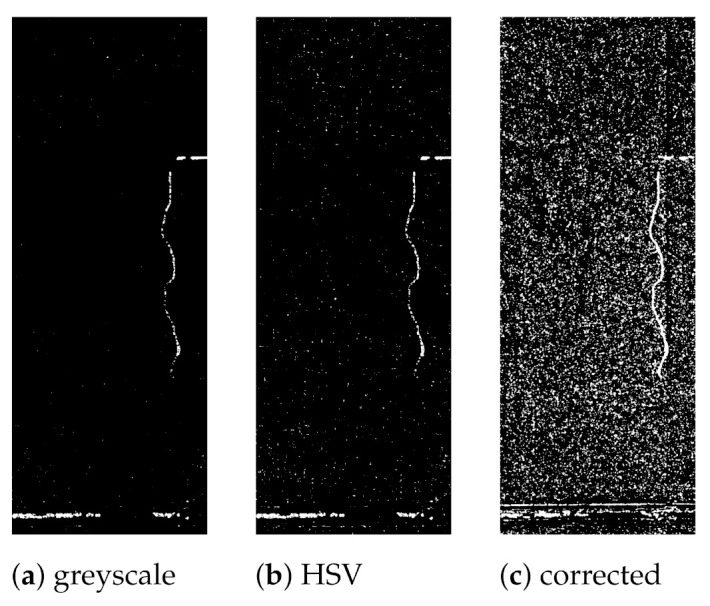
Change maps from SSIM. Greyscale (**a**), HSV (**b**) and corrected (**c**).

Detection of changes between two images can be considered as a comparison of similarities between two images [35]. The larger the difference between the survey image and the reference image is, the smaller the SSIM will be. This metric is based on the human visual system and is in line with what humans perceive as change. SSIM [36] performs different similarity measurements of luminance, contrast and structure, and thereafter combines them to obtain a single value. Considering two image blocks *A* and *B*, the SSIM is given by:

(6) $$SSIM\left(A,B\right)=\frac{\left(2\mu_{A}\mu_{B}+c_{1}\right)\left(2\sigma_{AB}+c_{2}\right)}{\left({\mu_{A}}^{2}+{\mu_{B}}^{2}+c_{1}\right)\left({\sigma_{A}}^{2}+{\sigma_{B}}^{2}+c_{2}\right)}$$

where $\mu_{A}$, $\mu_{B}$ are the mean and ${\sigma_{A}}^{2}$, ${\sigma_{B}}^{2}$ are the variance of *A* and *B* while $\sigma_{AB}$ is the covariance between *A* and *B*. Constants $c_{1},c_{2},c_{3}$ are calculated using:

(7) $$c_{1}={\left(K_{1}L\right)}^{2},\;c_{2}={\left(K_{2}L\right)}^{2},\;c_{3}=\frac{c_{2}}{2}$$

where $K_{1},K_{2}\ll 1$, generally $K_{1}=0.01,K_{2}=0.03$ as set in [36] and *L* is the dynamic range of the pixel values (L=255 for 8-bit greyscale images). Here, SSIM is used as a PBCD method to generate a CM between a reference and survey image. Therefore, considering the neighbourhood around point (x,y) in two temporal images $I(x,y,t_{1})$ and $I(x,y,t_{2})$ then the SSIM at point (x,y) is calculated using Equation (6). The SSIM is normalised to a range of [0,255] and thresholded using:

(8) $$D\left(x,y\right)=1-\frac{SSIM(x,y)+1}{2}$$

(9) $$CM\left(x,y\right)=\left\{\begin{array}{cc} 1 & \mathrm{if}\;D(x,y)\geq \;T\\ 0 & \mathrm{otherwise} \end{array}\right.$$

where D(x,y) represents the difference image and *T* is a constant. A fixed threshold value cannot satisfy all scenarios; thus, the Gaussian VE automatic thresholding method is applied. An investigation of the performance in change detection is done using greyscale images, the V channel in HSV images and pre-processed images corrected for uneven illumination. In general, the best results with minimum noise were obtained using greyscale images, as shown in Figure 10.

## 7. Decision-Level Fusion

Considering the complementary advantages of the implemented PBCD methods, the generated CMs from image differencing ($CM_{diff}$), PCA ($CM_{PCA}$) and SSIM ($CM_{SSIM}$) are fused into a single CM using decision-level fusion methods. Different decision-level fusion methods were implemented; however, the PCA-weighted sum and the majority voting gave the best results and are reported in this section.

### 7.1. PCA-Weighted Sum

**Figure 11:**
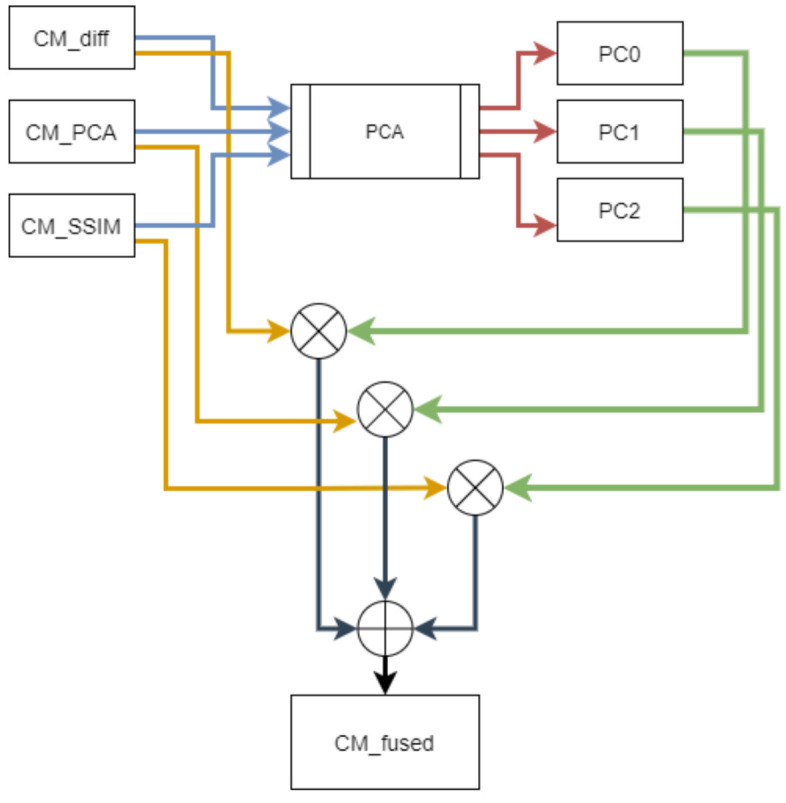
Diagram of change map fusion by PCA.

The PCA-based fusion algorithm is illustrated in Figure 11, where PC0,PC1, and PC2 are the first three components of one PCA analysis of the stacked CMs. The first three principal components are used as they represent most of the variation in the data. The *PCA* is applied on the three CMs, namely $CM_{D},CM_{PCA}$ and $CM_{SSIM}$. The first principal component, $C_{0}$, measures mainly the impact of the difference CM, the second principal component, $C_{1}$, has a strong association with PCA CM and the third principal component, $C_{2}$, more so represents the SSIM CM. Hence, these $C_{i}$ are used as weights of the CMs to give more importance to the CMs that have better captured the change. The summation of these weighted terms generates the fused *CM* using:

(10) $$CM_{PCA}=CM_{D}\cdot C_{0}+CM_{PCA}\cdot C_{1}+CM_{SSIM}\cdot C_{2}$$

**Figure 12:**
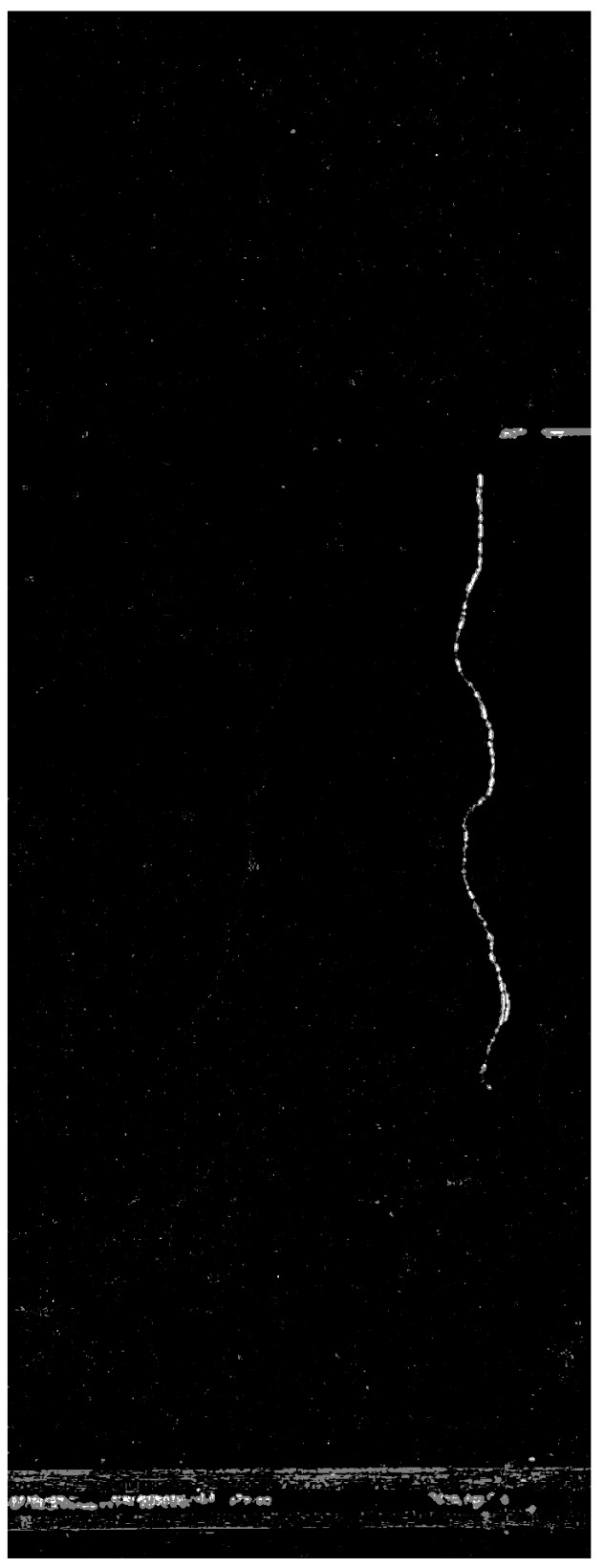
Change map fusion by PCA.

As shown in Figure 12, the PCA approach generates few noise pixels while retaining the actual changes; in this case, those belonging to the crack.

### 7.2. Majority Voting

**Figure 13:**
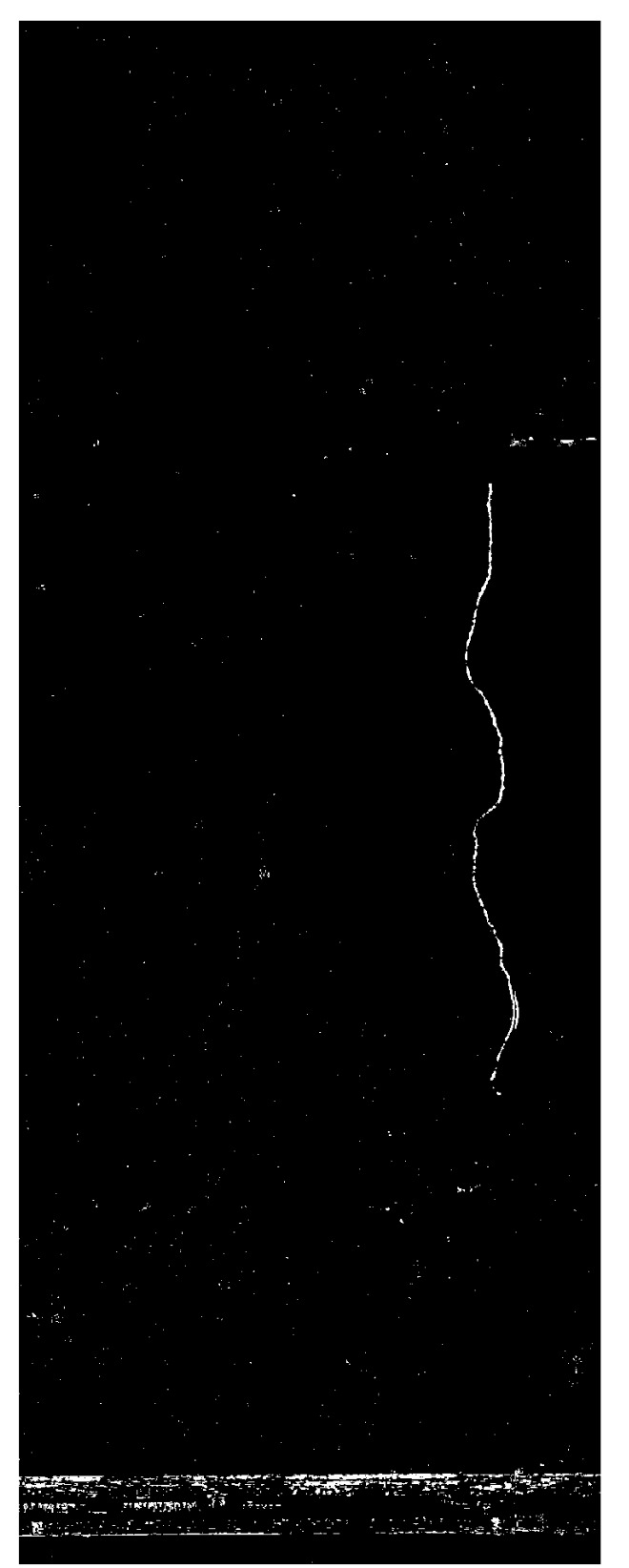
Change map fusion by majority voting.

In the majority voting algorithm, the three different CMs ($CM_{D}$, $CM_{PCA}$ and $CM_{ssim}$) cast a unit vote and if at least two of the CMs register a change, then the corresponding pixel in the fused CM is assigned 1 (change), otherwise 0 (no change). Similar to the previous method, this fusion approach generates only a few noise pixels while retaining the actual changes, as shown in Figure 13.

## 8. Change Map Analysis

**Figure 14:**
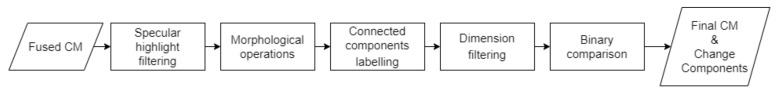
Change map analysis process.

At this point, the fused CM may still contain “nuisance change” areas that should not be considered as “changes”. Hence, the CM analysis process illustrated in Figure 14 was developed.

### 8.1. Specular Highlights Filtering

Fusion between the inverse specular highlight mask image SpecH(x,y) and the final $CM_{MV}\left(x,y\right)$ is done through an AND operation defined by:

(11) $$CM_{filtered}\left(x,y\right)=CM_{MV}\left(x,y\right)\wedge SpecH\left(x,y\right)$$

### 8.2. Morphological Operations

The filtered fused $CM_{filtered}\left(x,y\right)$ may contain some small “change areas” coming from image noise and minor registration errors. Here, a morphological closing operation that uses dilation and erosion sequentially, is applied to the fused CM. This joins any change segments by filling gaps, such as in “crack changes”, while at the same time ignores the “noise changes”.

### 8.3. Connected Components Labelling

Next, connected components labelling with 8-connectivity is used to identify and group neighbouring pixels into “change components”.

### 8.4. Dimension Filtering

The components are now filtered by their size. A “change component” is only retained if its width and/or height satisfies the corresponding thresholds $T_{W},T_{H}$. Using the GDAL library [37], the orthophoto raster scale is obtained, and using the simple proportion principle, the physical dimensions of the segment’s field of view (FoV) are calculated. Using the configurable parameter $d_{min}$ representing the minimum dimension for a detected change together with the corresponding image dimension and FoV, the thresholds are calculated using:

(12) $$T_{W}=\frac{d_{min}\times W}{FoV_{W}}$$

(13) $$T_{H}=\frac{d_{min}\times H}{FoV_{H}}$$

where *W* and *H* represent the width and the height of the image, respectively.

If a candidate “change component” has a width larger than $T_{W}$ and/or a height larger than $T_{H}$ then the component is confirmed as a “change component”.

### 8.5. Binary Comparison

A further analysis is done to reduce false changes due to reflections, shadows and parallax errors. The images consist of a white background and darker areas where cracks and marks, etc., appear. First, the images are inverted, then the bounding rectangle of each “change candidate” is masked out of both the reference and survey images using the corresponding area in the CM as a mask. The difference in the number of pixels is divided by the total number of mask pixels.

**Figure 15:**
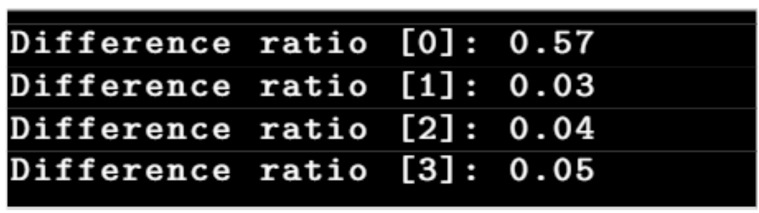
Difference ratios of “change candidates”.

**Figure 16:**
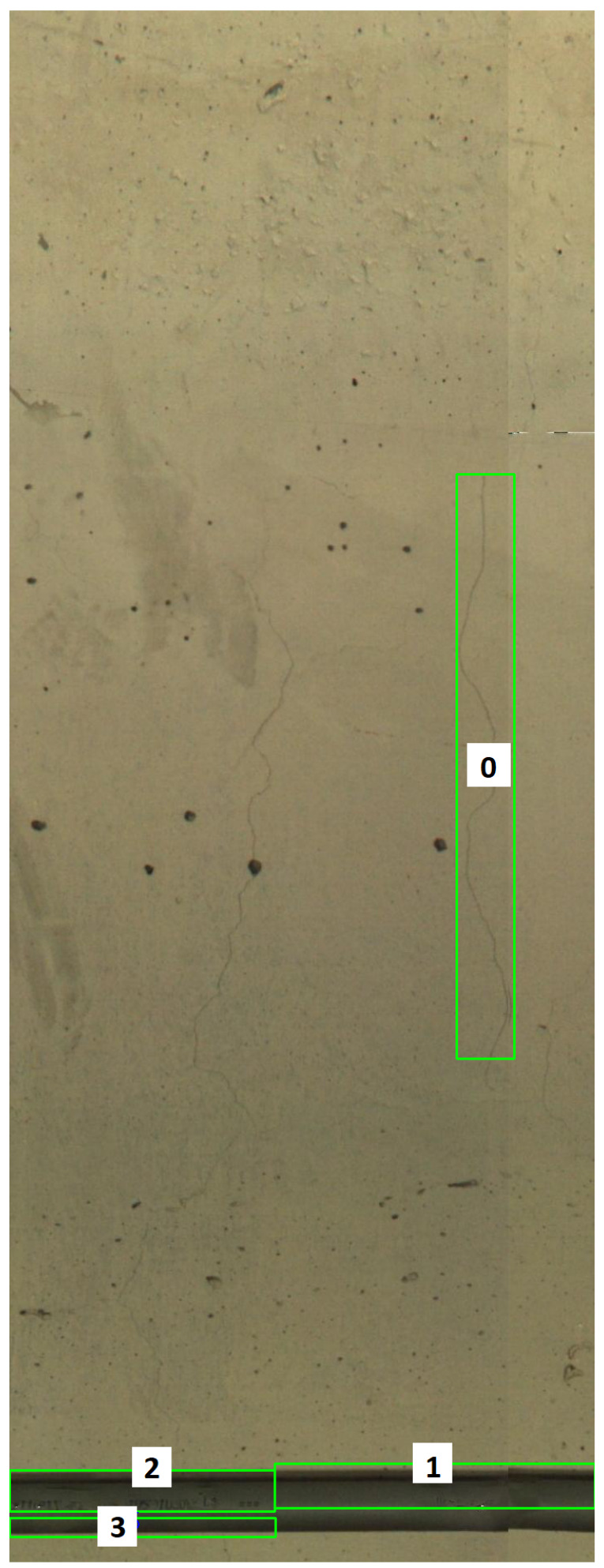
The change candidates corresponding to the example in Figure 6.

**Figure 17:**
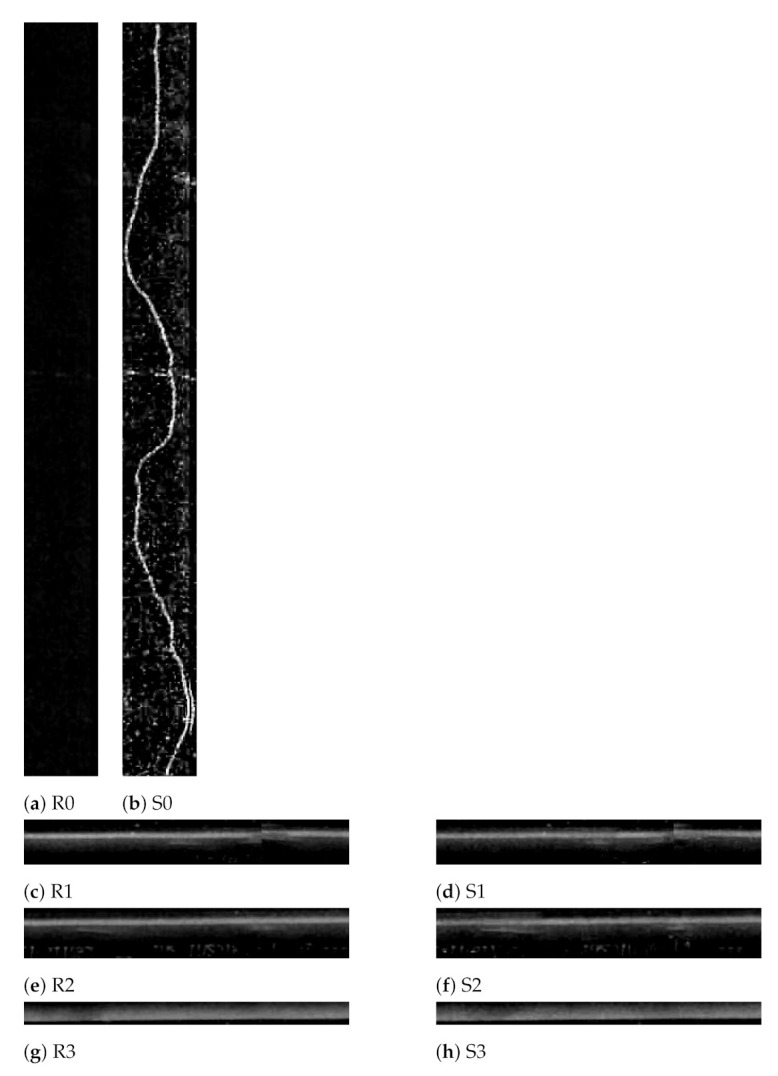
Change candidates (**a**,**b**) reference and survey patch ‘0’, (**c**,**d**) reference and survey patch ‘1’, (**e**,**f**) reference and survey patch ‘2’ and (**g**,**h**) reference and survey patch ‘3’.

Considering the same example, the difference ratios given in Figure 15 correspond to the “change candidates” shown in Figure 16, whose image patches are displayed in Figure 17. This shows that the difference ratio for component 0, which is the “actual change”, is much larger than for the others. Thus, a threshold is empirically set to filter out the “false changes”. If the ratio is higher than a threshold, this is considered as a “change”; otherwise, it is ignored such that in this case, for example, only “change candidate 0” is considered as a change.

## 9. Performance Evaluation

To demonstrate the effectiveness of the proposed change detection module, a set of experiments were conducted by simulating different changes such as cracks and other markings on the walls. The experiment covers 60 m of the LHC tunnel. In addition, some markings were also made on the images during post-processing, using a graphical editing software.

For each test scenario, the changed areas are manually marked with a red dot. The change detection output marked with green boxes and indices is analysed and manually compared to the corresponding reference-survey image pair.

An actual “change component” is marked as a true positive (TP). Each actual “change component” that is not detected by the algorithm is added to the false negative (FN) list. On the other hand, an area that is falsely detected as a change, as it does not correspond to any of the actual changes, is noted as a false positive (FP). To quantitatively evaluate the performance of the change detection algorithm, the following metrics are used.

### 9.1. Evaluation Metrics

The recall is calculated using the true positive rate (TPR), implying the system’s ability to find the changes. The precision is calculated using the positive detection rate (PDR), implying the system’s ability to identify only the actual changes. The F1−score is also calculated to find an optimal blend of both. These metrics are found using:

(14) $$TPR\left(Recall\right)=\frac{TP}{TP+FN}\times 100\%$$

(15) $$PDR\left(Precision\right)=\frac{TP}{TP+FP}\times 100\%$$

(16) $$F1-score=2\times \frac{Precision\times Recall}{Precision+Recall}\times 100\%$$

### 9.2. Quantitative Analysis

The quantitative results recorded in Table 1 show that the decision-level fusion by PCA generated a higher precision rate. As the threshold of the final binary comparison was increased from 0.1 to 0.2, the precision value increased from 83.0% to 94.5%. When the majority voting approach was used, precision of 78.8% and 93% was achieved at the same thresholds of 0.1 and 0.2 in the final comparison stage. This implies that the PCA approach distinguished better between actual and nuisance changes.

However, it is also important to evaluate the effectiveness of the algorithm with respect to its ability to find all the data points of interest, in this case, the identified changes. This is given by the recall rate, which had higher values of 83.71% and 81.11% for the majority voting approach with binary comparison stage threshold values of 0.1 and 0.2, respectively. This implies that the majority voting approach could identify more actual changes with fewer misses.

It is beneficial if the algorithm can correctly classify the changes to avoid false alarms; however, it is important that changes due to defects on the tunnel lining are not missed. Hence, a trade-off between precision and recall is essential. This is found by analysing the F1−score, which combines both metrics. As observed in Table 1, the fusion using a majority voting approach achieved better general performance with respect to the F1−score.

### 9.3. Qualitative Analysis

In addition to the quantitative results, a qualitative analysis was made on different scenarios with “crack changes”, other defects and also “nuisance changes” caused by varying light conditions and shadows.

**Figure 18:**
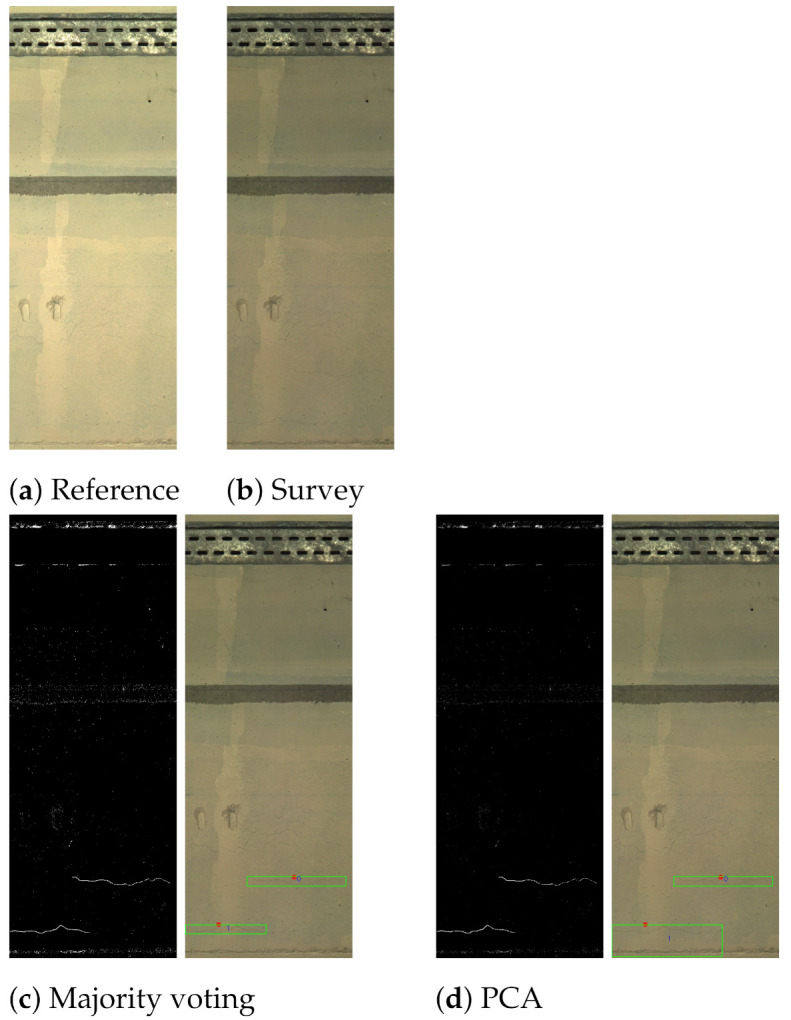
An example showing similar results for both majority voting and PCA. Reference (**a**), survey (**b**), majority voting (**c**) and PCA (**d**).

In the example presented in Figure 18, both of the fusion approaches identified the actual changes correctly. However, the majority voting approach gave a more confined bounding box around the “crack change” labelled 1.

**Figure 19:**
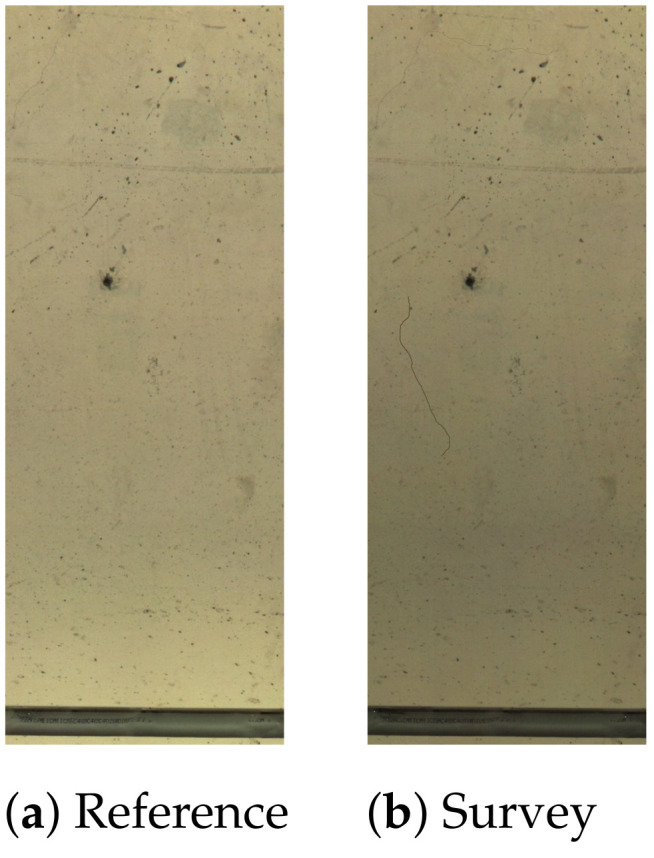

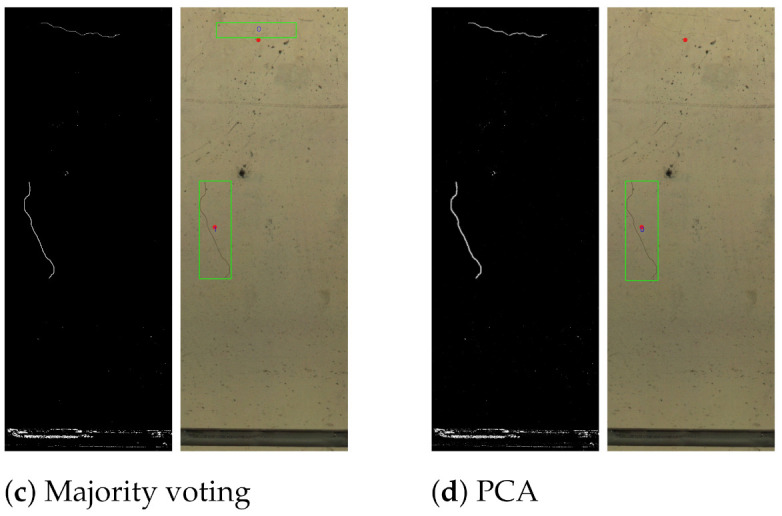
An example showing different detection results from majority voting and PCA. Reference (**a**), survey (**b**), majority voting (**c**) and PCA (**d**).

Using the reference and survey images in Figure 19, the change detection algorithm using majority voting correctly identified both of the “crack changes”; however, the connectivity and binary comparison stages following the PCA method incorrectly identified this as a “nuisance change” and, thus, discarded it.

**Figure 20:**
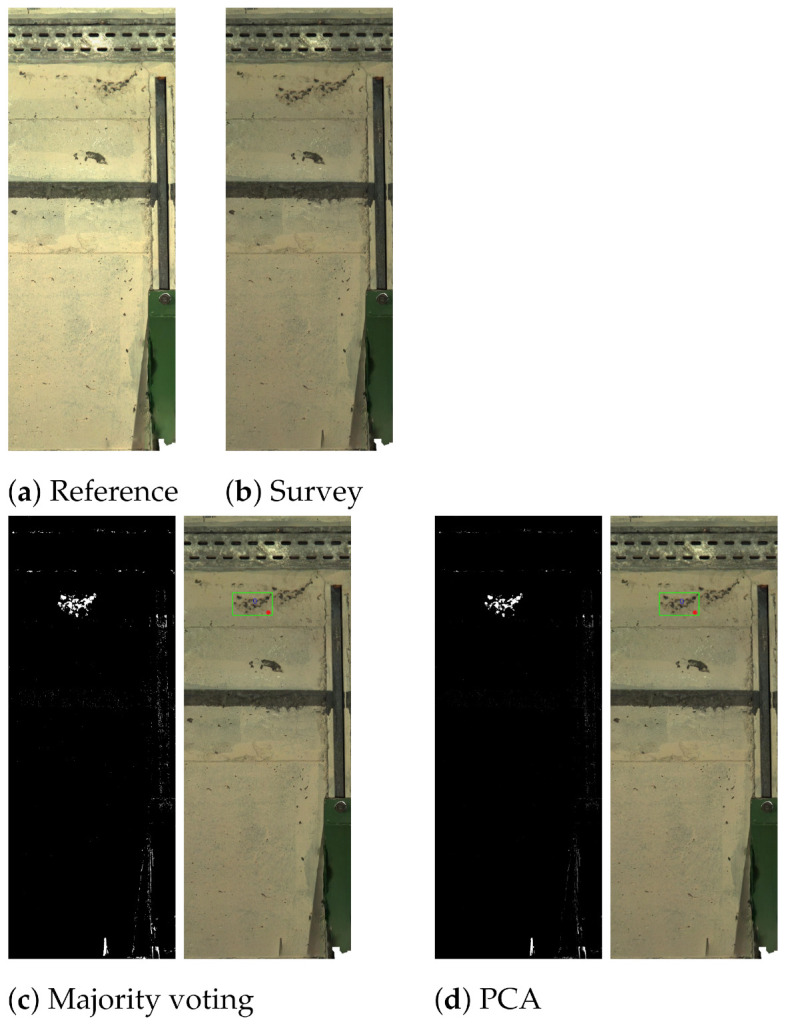
An example showing similar performance of majority voting and PCA solutions on a different simulated defect on the wall. Reference (**a**), survey (**b**), majority voting (**c**) and PCA (**d**).

**Figure 21:**
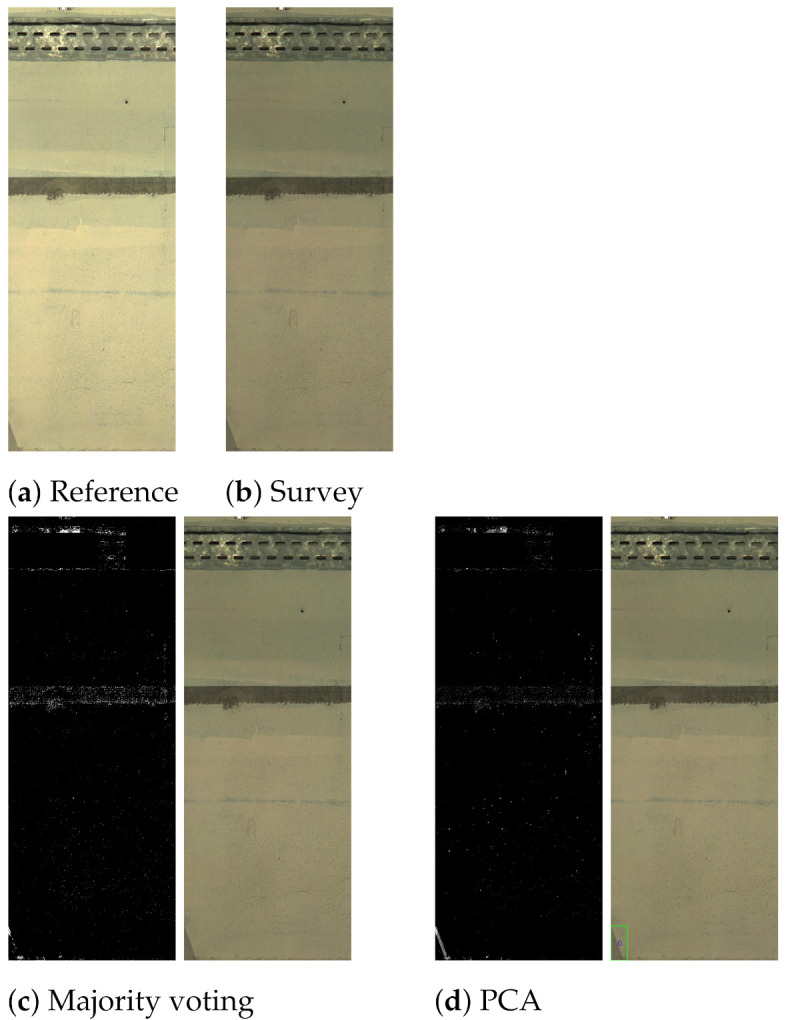
An example exhibiting lighting changes that are correctly identified as a nuisance and not detected as a change. Reference (**a**), survey (**b**), majority voting (**c**) and PCA (**d**).

In Figure 20, another “defect” was simulated on the wall. In this case, both methods correctly identified the change. The final example in Figure 21 only exhibits “nuisance changes” with respect to the light. Both CMs show white pixels in different areas in the image, implying possible change due to specular highlights, shadows and light changes. However, the CM analysis stage ignored most of these regions except for the small shadow area at the bottom of the image when using PCA-based fusion, generating a “false change”.

Considering both the quantitative and qualitative results, the majority voting approach for the decision-level fusion while using a threshold of 0.2 for the final binary comparison stage is used for the final implementation of the vision-based tunnel lining change detection system.

## 10. Conclusions

Periodic tunnel structural monitoring and inspection are a necessity. Inspections are predominantly performed through visual observations, which involve looking for structural defects and making sketches for civil engineers to assess them and, in turn, suggest the required maintenance and/or repairs. Associated with this, there are several drawbacks, including personnel exposure to hazardous conditions and outcome subjectivity that is highly dependent on human intervention, which may lead to inaccuracies or misinterpretations. Considering this, a novel tunnel inspection solution to monitor for changes on tunnel linings was proposed. An automatic image data acquisition system integrated on a robotic platform is used to capture tunnel wall images. To alleviate the impact of different light conditions on the change detection algorithm, pre-processing stages were also implemented. These include a shading correction to adjust uneven illumination and highlights localisation to reduce false changes due to flashlight reflections. Subsequently, a new change detection algorithm was developed through a combination of different bi-temporal pixel-based fusion methods and decision-level fusion of change maps. The proposed solution complements current structural health monitoring techniques and provides a better means of tunnel surface documentation.

While providing a step forward, our future work will focus on the improvement of the change detection algorithm and pre-processing stages. Machine learning solutions are producing promising results in the field of computer vision, and such solutions can be added to or replace this change detection algorithm. However, a much larger dataset will be required to train and test such solutions.

## Figures and Tables

**Table 1:** Quantitative results from the change detection algorithm.

Fusion Method	TH	TP	FP	FN	TPR %	PDR %	F1-Score %
MV	0.1	149	40	29	83.7	78.8	81.0
MV	0.2	146	11	34	81.1	93.0	**86.7**
PCA	0.1	137	28	39	77.8	83.0	80.4
PCA	0.2	103	6	73	58.5	94.5	72.3

## Data Availability

Some or all data, models, or code that support the findings of this study are available from the corresponding author upon reasonable request.
